# Reduction of intracerebral hemorrhage in hemodialysis patients after reducing aspirin use: A quality-assurance observational study

**DOI:** 10.1371/journal.pone.0185847

**Published:** 2017-10-02

**Authors:** Mabel Aoun, Sahar H. Koubar, Leony Antoun, Hani Tamim, Maha Makki, Dania Chelala

**Affiliations:** 1 Department of Nephrology, Saint-Georges Hospital, Ajaltoun, Lebanon; 2 Department of Nephrology, Saint-Joseph University, Beirut, Lebanon; 3 Department of Internal Medicine, Division of Nephrology, American University of Beirut Medical Center, Beirut, Lebanon; 4 Department of Internal Medicine, Holy Spirit University, Kaslik, Lebanon; 5 Biostatistics Unit, Clinical Research Institute, American University of Beirut Medical Center, Beirut, Lebanon; Hospital Universitario de la Princesa, SPAIN

## Abstract

There is so far no international consensus concerning the prescription of antithrombotic agents in hemodialysis patients. It is not clear yet why they cause more bleeding in some patients and are beneficial in others. We therefore tried to find out what triggers bleeding in this population. This is an observational before-and-after study that included all patients undergoing hemodialysis in our center between 2005 and 2015. We divided the study into two phases: phase one (125 patients) where aspirin was used without restrictions and phase two (110 patients) where aspirin was avoided in severe hypertension and primary prevention. We aimed to assess the differential occurrence of intracerebral hemorrhage between the two phases and the cardiovascular mortality of patients whether on aspirin or not. Bleeding events occurred in 12.8% of patients in phase one and 13.6% in phase two (*p* = 0.85). Seven out of 125 patients (6%) in phase one experienced intracerebral hemorrhage and none in phase two. Intracerebral hemorrhage was significantly increased in those with the combination of aspirin and severe hypertension (*p* = 0.003). Aspirin and acenocoumadin were significantly associated with total bleeding (OR = 3.81 and 4.85 with *p* = 0.005 and 0.001 respectively). Cardiovascular mortality did not differ between phase one and two whether patients were on aspirin or not (*p* = 0.45 and 0.31 respectively). Minimizing aspirin use in hemodialysis patients with severe hypertension reduced intracerebral bleeding without a significant difference in cardiovascular mortality.

## Introduction

Aspirin is a well-established treatment for secondary prevention of cardiovascular disease (CVD) in the general population [1]. It is recommended to reduce myocardial infarction, stroke and vascular death risks [1,2]. However, as a primary prevention, the evidence for aspirin is not clarified and even not justifiable [3]. A recent meta-analysis, that studied aspirin for primary prevention, revealed a reduced risk for nonfatal myocardial infarction (MI) but not for nonfatal stroke. Furthermore, it showed no benefit for all-cause or cardiovascular mortality [4]. Along with this uncertainty of reducing mortality in some categories of patients, aspirin’s main complication is bleeding. The incidence of major bleeding events seems to be five times higher in aspirin users [5].

Extrapolating from the general to the hemodialysis (HD) population is debatable because aspirin use in end-stage renal disease (ESRD) patients may be further associated with major bleeding events such as gastrointestinal bleeding (GIB) and intracerebral hemorrhage (ICH) [6,7]. However, cardiovascular mortality in patients on dialysis is 10 to 20 times higher than in the general population and it is due to both traditional and non-traditional cardiovascular risk factors [8,9]. Consequently, oral antiplatelet agents (aspirin or/and clopidogrel) are frequently prescribed in this population but the evidence behind their use is conflicting and the indications are still not well defined [6,10–13]. Based on the 2005 KDOQI Clinical Practice Guidelines for Cardiovascular Disease in Dialysis Patients, there were no sufficient trials in this population that can establish the safety and efficacy of aspirin except following MI [14].

There are also conflicting data about oral anticoagulant use in dialysis. Their indication for stroke prevention in patients with atrial fibrillation is well recognized and approved in the general population [15]. However in dialysis patients, they may be prescribed for atrial fibrillation or vascular access patency (VAP) but with a high risk of bleeding or calciphylaxis [16,17].

Obviously, until now, there is no general agreement concerning the prescription of antithrombotic agents especially aspirin in the HD population. It is not clear yet why they cause more bleeding in some patients and are beneficial in others [13]. The present paper seeks to understand the factors that may trigger bleeding in HD patients treated with antithrombotic agents. It is an observational study divided into two phases: phase one where aspirin was given to patients as per their cardiologists’ recommendation and phase two where aspirin was no more prescribed as a primary prevention and mostly avoided in severely hypertensive patients. The objective of this study is to find out whether minimizing aspirin use in hemodialysis patients would reduce bleeding events without affecting cardiovascular mortality.

## Materials and methods

### Study design, setting and participants

This is an observational before-and-after study (quality-assurance study) where the intervention was minimizing aspirin use. All patients with ESRD undergoing chronic HD in Saint-Georges Hospital dialysis unit, between January 2005 and December 2015, were included in this study.

The study was divided into two phases: 1-phase one (before intervention) between January 2005 and December 2011, 2- phase two (after intervention) between January 2012 and December 2015.

The reason for this study design is the new policy that was introduced at our center at the end of 2011, concerning antithrombotic agents’ prescription, following several episodes of ICH. In phase two, we stopped prescribing aspirin as a primary prevention. We allowed antiplatelet agents for patients with previous myocardial infarction, coronary stents and/or peripheral artery disease. As a secondary prevention, aspirin was prescribed on a daily basis if blood pressure was controlled or on alternate day basis or replaced by clopidogrel if they had severe uncontrolled hypertension. Oral anticoagulants were prescribed in any case of heart valve replacement, atrial fibrillation or a previous vascular access thrombosis. All patients were dialyzed three times per week.

Any patient who was transplanted or shifted to peritoneal dialysis was followed till the day he was out of HD.

There was no change in other clinical practices between the two phases of the study.

This study was approved by the ethics committee of Saint-Joseph University (CEHDF 860). The ethics committee waived the requirement for informed consent of patients because data were collected retrospectively.

### Definitions and classifications

Antithrombotic agents include antiplatelet agents and oral anticoagulants. The antiplatelet medications used in our center were aspirin and clopidogrel. Aspirin was given at a dose of 100 mg / day. The oral anticoagulant was acenocoumadin. Patients were taking heparin during their sessions as a loading dose of 2500 or 5000 IU per session with no maintenance dose. Heparin and oral antithrombotic agents were stopped definitely if any major bleeding event occurred.

Hypertensive patients were defined as taking one or more antihypertensive medications or having a pre-dialysis systolic blood pressure > 140 mmHg for more than 2 sessions weekly. Severe uncontrolled hypertension was defined as a pre-dialysis systolic blood pressure above160 mmHg in more than two sessions weekly.

### Data collection

Data were retrospectively collected from the patients’ medical records on the following parameters: demographics (age and gender), dialysis vintage, smoking, diabetes, hypertension, severe hypertension, indications for antithrombotic agents (primary prevention, coronary artery disease (CAD), peripheral artery disease (PAD), stroke, atrial fibrillation, vascular access patency (VAP), deep venous thrombosis (DVT)), bleeding events, cardiovascular mortality (CVM) and all-cause mortality (ACM).

Bleeding events were divided into four subgroups: intracerebral hemorrhage (ICH), gastro-intestinal bleeding (GIB), pulmonary bleeding and hematuria.

### Statistical analysis

The Statistical Package for Social Sciences (SPSS), version 24.0 was used for data entry, management, and analyses. Continuous data were reported as means and standard deviation (±SD) and were compared between different groups using the independent Student's t-test. On the other hand, categorical data were reported as numbers and percentages and were compared using the Chi-Square test, or the Fishers exact test, as appropriate. Multivariate regression analysis was used to adjust for potentially confounding variables. Variables adjusted for were dialysis vintage, smoking, hypertension, diabetes, coronary artery disease, acenocoumadin, clopidogrel and aspirin. The stepwise logistic regression analysis assessed the association between total bleeding and the different predictors. *P*-value of 0.05 was set for the entry of potential predictors into the model, whereas a *p*-value of 0.1 was set for removal from the model. The results were presented by the odds ratio (OR) and 95% confidence interval (CI). *P*-value of < 0.05 was considered statistically significant.

## Results

### Baseline demographic and clinical characteristics

**Fig 1:**
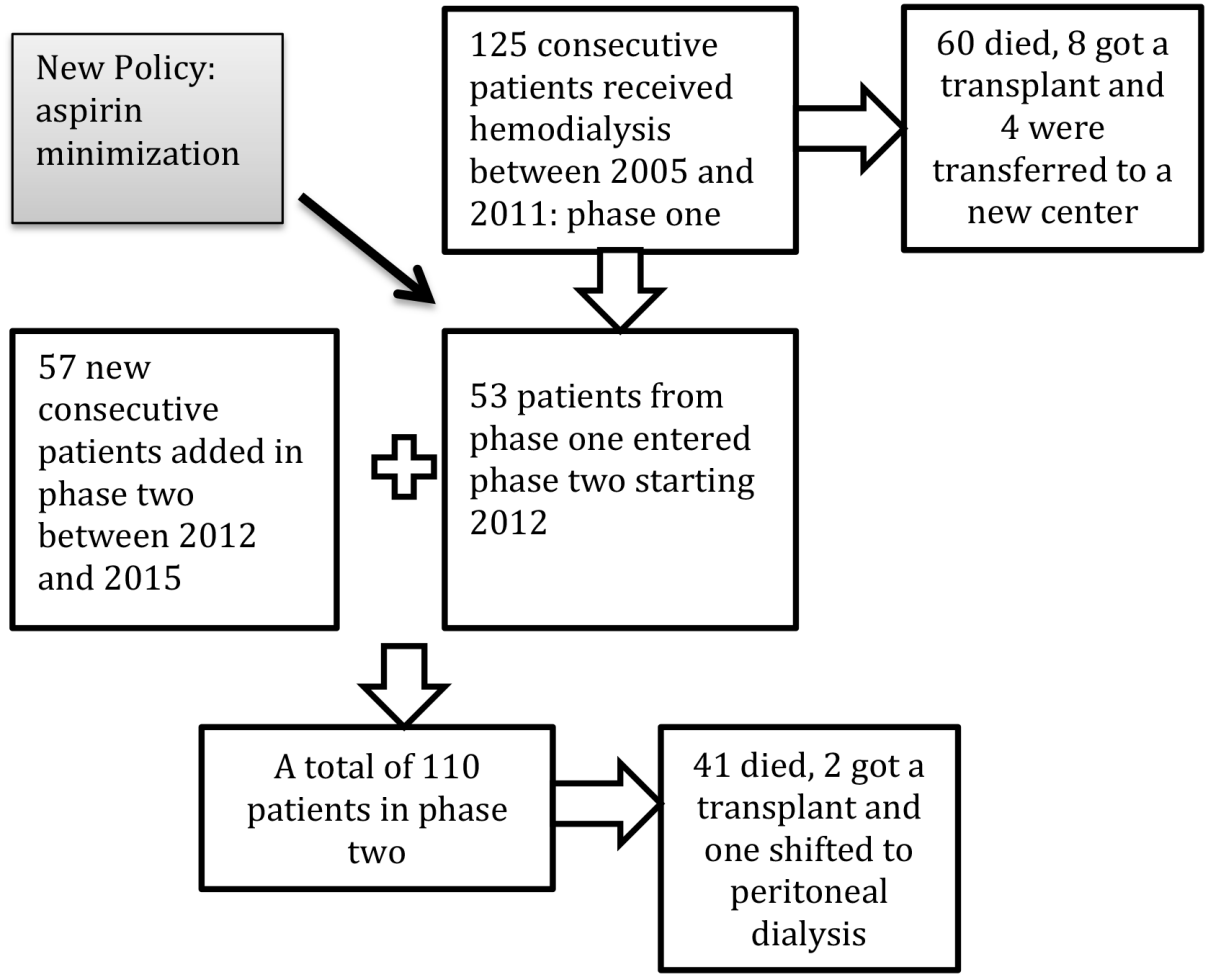
Flow diagram of the consecutive inclusion of all patients between January 2005 and December 2015.

Fig 1 shows the consecutive inclusion of a total of 182 patients and their follow-up: 125 patients in phase one and 110 patients in phase two.

Table 1 illustrates the demographic and clinical characteristics of all patients. All patients were dialyzed three times per week with a session duration average of 3.8 ±0.3 hours. All patients were white.

**Table 1:** Demographic and baseline clinical characteristics of patients in the two phases.

	Phase One N = 125	Phase Two N = 110	*P*-value*
**Age, years (mean ±SD)**	64.41 ± 14.90	66.61 ± 13.48	0.24
**Gender M/F (%/%)**	76/49 (60.8/39.2)	70/40 (63.6/36.4)	0.65
**Smoking, n (%)**	53 (42.4)	42 (38.2)	0.51
**Dialysis vintage, months (mean ±SD)**	41.96 ± 36.13	49.13 ± 44.41	0.18
**Diabetes, n (%)**	57 (45.6)	51 (46.4)	0.91
**Hypertension, n (%)**	84 (67.2)	95 (86.4)	0.001
**Severe hypertension, n (%)**	23 (18.4)	18 (16.4)	0.68
**Coronary artery disease, n (%)**	22 (17.6)	44 (40.0)	<0.0001

*Chi-square test, significant p-value < 0.05.

In total, 125 patients were included in phase one: age ranged between 15 and 90 years with a mean age at inclusion of 64.4 ±14.9 years. It was found that 18.4% were severely hypertensive. Six of those severe hypertensive patients were on daily aspirin.

110 patients were included in phase two: age ranged between 20 and 85 years with a mean age at the beginning of phase two of 66.6 ±13.5 years. Similarly, 16.4% were severely hypertensive. Two of those severe hypertensive patients were on alternate day aspirin and three were on clopidogrel.

### Antithrombotic agents: Distribution and indications

In phase one, 21% of the patients were on aspirin alone, 12% were on clopidogrel alone, 3.2% on DAT, 15.2% on acenocoumadin and 49.8% were not taking any antithrombotic agent. In phase two, 10% were on aspirin alone, 19.1% on clopidogrel alone, 9% on DAT, 10.9% on acenocoumadin and 50.9% on no antithrombotic agent (Table 2).

**Table 2:** Antithrombotic agents distribution in the two phases, n (%).

	Phase One N = 125	Phase Two N = 110	*P*-value*
**Acenocoumadin**	19 (15.2)	12 (10.9)	0.33
**Aspirin alone**	26 (20.8)	11 (10.0)	0.02
**Clopidogrel alone**	15 (12.0)	21 (19.1)	0.13
**Dual antiplatelet use**	4 (3.2)	10 (9.1)	0.16
**No antithrombotic agents**	61 (48.8)	56 (50.9)	0.75

*Chi-square test, significant p-value < 0.05.

Table 3 summarizes the different indications of any antithrombotic agent in the two phases. After considering each antithrombotic agent, we found out that, in phase one, 42.3% of patients on aspirin were taking it for CAD, 19.2% for PAD, 27% as primary prevention, 3.8% for atrial fibrillation and 3.8% for VAP. Among patients on clopidogrel, 40% of them were taking it for CAD, 20% for PAD, 33.3% for primary prevention and 6.7% for VAP. For patients on DAT, 50% of them were taking the combination for CAD, 25% for PAD and one patient for primary prevention. And finally among patients on acenocoumadin, 42.1% of them were taking the medication for VAP, 15.8% for atrial fibrillation and 26.3% for PAD. In phase two, 81.8% of patients on aspirin were taking it for CAD, 9.1% for PAD. 62% of patients on clopidogrel were taking it for CAD, 10% for PAD. 80% of patients on DAT were on the combination for CAD, 20% for PAD. Patients on acenocoumadin were taking it for VAP and atrial fibrillation (58.3% and 25% respectively). None was taking aspirin in phase two as primary prevention.

**Table 3:** Main indications for antithrombotic agents’ use, n (%).

	Phase One N = 125	Phase Two N = 110	*P*-value*
**Vascular access patency**	10 (13.5)	13 (18.6)	0.41
**Coronary artery disease**	20 (23.8)	30 (34.5)	0.12
**Stroke**	0 (0.0)	1 (1.7)	0.29
**Peripheral artery disease**	14 (17.9)	5 (8.1)	0.09
**Primary prevention**	13 (16.9)	0 (0.0)	0.001
**Atrial fibrillation**	4 (5.9)	3 (5.0)	1.00
**Deep venous thrombosis**	0 (0.0)	1 (1.7)	0.29

*Chi-square test, significant p-value < 0.05.

### Bleeding

We found that 12.8% of the patients in phase one and 13.6% in phase two experienced bleeding (Table 4 and S1 Table). ICH occurred in 6% of patients in phase one and none in phase two. When analyzing ICH events, we found a strong association between ICH and the combination of aspirin with severe hypertension in phase one (*p* = 0.003) (Table 5).

**Table 4:** Outcomes: Bleeding and death in the two phases.

		Phase One N = 125	Phase Two N = 110	*P*–value*
**Bleeding occurrence, n (%)**				
No		109 (87.2)	95 (86.4)	
Yes		16 (12.8)	15 (13.6)	0.85
Bleeding sites**, **n (%)**				
Digestive		8 (6.8)	13 (12.0)	0.18
Cerebral		7 (6.0)	0 (0.0)	0.02
Pulmonary		1 (0.9)	1 (1.0)	1.00
Bladder		0 (0.0)	1 (1.0)	0.47
**Death**				
No		65 (52.0)	69 (62.7)	
Yes		60 (48.0)	41 (37.3)	0.10
Death–Causes***, **n (%)**				
Tumor		5 (7.1)	3 (4.2)	0.49
Heart failure		0 (0.0)	2 (2.8)	0.50
Septic shock		7 (9.7)	6 (8.0)	0.71
Cardiac arrest		34 (34.3)	27 (28.1)	0.35
Cerebral bleeding		6 (8.5)	0 (0.0)	0.01
Total bleeding		9 (12.2)	1 (1.4)	0.02
Stroke		3 (4.4)	0 (0.0)	0.12
All Cardiovascular Death	All sample	45 (40.9)	31 (31.0)	0.14
	On aspirin	13 (54.2)	4 (40.0)	0.45
	Not on aspirin	32 (37.2)	27 (30.0)	0.31

*Chi-square test, significant p-value < 0.05.

**The reference is those who did not bleed.

***The reference is those who did not die

**Table 5:** Subanalysis of cerebral hemorrhage in severe hypertensive patients before and after aspirin reduction.

**Phase I (N = 23)**		On aspirin N = 5	Without aspirin N = 18	*P*-value*
	**Cerebral Hemorrhage**			0.003
	No (%)	1 (20.0)	17 (94.4)	
	Yes (%)	4 (80.0)	1 (5.6)	
				
**Phase II (N = 18)**		On aspirin N = 2	Without aspirin N = 16	*P*-value*
	**Cerebral Hemorrhage**			Non-estimable
	No (%)	2 (100.0)	16 (100.0)	
	Yes (%)	0 (0.0)	0 (0.0)	

*Chi-square test, significant p-value < 0.05.

Factors significantly associated with total bleeding were aspirin and acenocoumadin (*p*-value 0.005 and 0.001 respectively). Clopidogrel was not associated with increased bleeding (Table 6).

**Table 6:** Factors associated with total bleeding events.

	**Total bleeding events**		***P* -value**
	**No** **N = 204**	**Yes** **N = 31**	
**Age, mean (±SD)**	64.91 ± 14.42	68.94 ± 12.86	0.14^§^
**Dialysis vintage, mean (±SD)**	45.33 ± 40.87	45.23 ± 36.87	0.99^§^
**Smoking, n (%)**	85 (41.7)	10 (32.3)	0.32^¥^
**Hypertension, n (%)**	157 (77.0)	22 (71.0)	0.47^¥^
**Coronary artery disease, n (%)**	56 (27.5)	10 (32.3)	0.58^¥^
**Diabetes, n (%)**	93 (45.6)	15 (48.4)	0.77^¥^
**Aspirin, n (%)**	28 (13.7)	9 (29.0)	0.03^¥^
**Acenocoumadin, n (%)**	22 (10.8)	9 (29.0)	0.01^¥^
**Clopidogrel, n (%)**	33 (16.2)	3 (9.7)	0.35^¥^

Multivariate analysis*			
	**OR (95% CI)**		***P* -value**
**Acenocoumadin**	4.85 (1.85–12.66)		0.001
**Aspirin**	3.81 (1.49–9.75)		0.005

*Variables included in the model were: age; dialysis vintage; smoking; hypertension; diabetes; coronary artery disease; acenocoumadin; clopidogrel; aspirin

^§^Student’s test for continuous variables,

^¥^Chi-square test for categorical variables, significant p-value < 0.05.

### Death

All-cause mortality in phase one and phase two was estimated at 48% and 37% respectively. S2 Table shows the yearly mortality percentage. Table 4 summarizes the different causes of death. Cardiovascular mortality did not differ between the two phases whether patients were on aspirin or not.

## Discussion

The most remarkable result emerging from our data analysis is the strong association between ICH and uncontrolled hypertension in patients on aspirin. After reducing aspirin use in severely hypertensive patients in the second phase of the study, the rate of ICH dropped to zero. To the best of our knowledge, this is the first time that the severity of hypertension is studied in HD patients with ICH on aspirin. A previous study probably concurs with our results. It compared ICH between HD and non-HD patients and showed that the lack of antihypertensive drugs in HD patients was a risk factor for ICH and mortality [18]. The only study (Robinson et al) that previously analyzed pre-dialysis blood pressure levels in HD patients showed that compared with the reference category 130–139 mm Hg, there was no consistent difference in mortality [19]. Their results concerning mortality are similar to ours, however, they did not analyze bleeding nor the concomitant use of antithrombotic agents. It is already known that, in the general population, ICH is a fatal complication of antithrombotic therapy and few authors recommend careful blood pressure management when prescribing these drugs [20–22]. Our findings support this recommendation in HD patients as well.

Another important finding of our study is the absence of difference in cardiovascular mortality between phase one and phase two whether patients were on aspirin or not. This conclusion is in good agreement with other studies that showed that aspirin tends to increase bleeding in ESRD patients without any proven constant beneficial effect on cardiovascular outcomes [6,7]. A recent study of 406 patients on regular HD with a 5-y follow-up showed that the cumulative survival rate was not significantly higher in the aspirin versus non-aspirin users. However, compared to our study, they did not find an increased risk of fatal cerebral hemorrhage in the aspirin users [7]. On the other hand, in a Canadian cohort, aspirin was prescribed in 38% of patients on HD and was significantly associated with higher mortality [12]. It could be argued that they had more patients with CAD and thus were more likely to be prescribed aspirin. However, they did not analyze the cause of death nor the degree of hypertension in those patients. In contrast to those above-mentioned non-beneficial effects of aspirin, there are other publications that support the use of aspirin. In 1998 a meta-analysis of 16 RCTs showed that aspirin was strongly associated with intracerebral hemorrhage but the overall benefit of aspirin use on myocardial infarction and ischemic stroke may outweigh its adverse effects [23]. However once more the degree of hypertension in those individuals was not analyzed and the meta-analysis did not target ESRD patients. Aspirin was shown to be an effective treatment for prevention of a second ischemic stroke in patients undergoing dialysis [11]. However secondary prevention of ischemic stroke with aspirin was not analyzed in our study because we had no use of aspirin for this indication.

Overall, results regarding aspirin in the literature are conflicting. For instance some data analysis from the Dialysis Outcomes and Practice Patterns Study (DOPPS) in 2007 showed that aspirin was significantly associated with decreased risk of stroke but increased risk of myocardial infarction and no increase in gastrointestinal bleeding [6]. In a review of clinical trials and cohorts studying antiplatelet agents in ESRD, Hiremath et al emphasized the variability and contradiction of outcomes in studies using aspirin [13]. From our point of view, this variability in outcomes could be explained by the fact that severe hypertension might have been a confounding variable that has been forgotten or poorly assessed. Given that hypertension is largely dependent on the volume overload of HD patients and often difficult to control, the best way to prevent intracerebral bleeding in those patients might be through avoiding antithrombotic agents.

In contrast to patients on aspirin, patients of our study on clopidogrel did not show an increase in the bleeding risk. This result is in good agreement with the findings of Holden et al where 34 patients had been treated with clopidogrel for a total of 46 person-years of exposure without a bleeding event [24]. In the Dialysis Access Consortium (DAC) study also, there was no difference in the bleeding risk between those on clopidogrel versus placebo [25]. Apparently, the bleeding risk differs among different antiplatelet agents and seems lower with clopidogrel. A good explanation of the low rate of bleeding with clopidogrel in CKD patients has been evaluated by Htun et al who showed a low responsiveness to clopidogrel by ADP-induced platelet aggregation in patients with stage 3 to 5 CKD [26].

When it comes to the combination of aspirin and clopidogrel, this strategy seems to increase the bleeding rate in the general population [27] and to be only appropriate for patients with acute coronary syndromes or recent vascular stenting but not in secondary prevention of stroke [2,28]. Moreover, the risk of bleeding in HD patients is more pronounced with the DAT as has been shown by Kaufman et al in their study about prevention of arteriovenous graft (AVG) failure [29]. In our study, the DAT use was minimal in the first phase of the study and this may explain the absence of ICH in those patients. In phase two, DAT use has increased because cardiologists started to use drug-eluting stents for coronary artery stenosis in dialysis patients but none of the patients on DAT was severely hypertensive. The latter could be a reason for the non-occurrence of ICH with DAT.

Another antithrombotic agent analyzed in our study was acenocoumadin. Oral anticoagulants are mainly used in HD patients for atrial fibrillation and VAP but without any proven beneficial effect on primary graft patency [30]. Thus the European Renal Best Practice (ERBP) guidelines on vascular access in HD in 2007 made no recommendations on anticoagulants for VAP but emphasized the increase rate of bleeding with warfarin [16]. Despite those recommendations, almost half of our patients on acenocoumadin were taking it to prevent vascular access thrombosis and experienced significant increase in bleeding. Regarding atrial fibrillation and oral anticoagulant in HD, several cohorts were published using data from the DOPPS to evaluate bleeding and stroke. First in 2010 warfarin use in HD patients aged older than 75 years has been associated with higher stroke risk, perhaps due to hemorrhagic stroke, but this could not be confirmed [31]. Then in 2013, they studied HD patients with atrial fibrillation on different antithrombotic agents and found out that rates of major bleeding substantially exceeded rates of stroke for all patient groups, even those at high stroke risk [32]. Oral anticoagulant in our patients was significantly associated with excessive bleeding mainly GIB. There was no ICH in the group taking acenocoumadin probably because the majority of those patients were not severely hypertensive. On the contrary they were relatively hypotensive. Therefore we assume that patients on acenocoumadin might have experienced ICH similarly to those on aspirin if they were severely hypertensive.

Finally, the only independent predictors for total bleeding in our study were aspirin and acenocoumadin. Aspirin led mainly to ICH and acenocoumadin to GIB. Curiously some previous studies have not found antithrombotic agents as an associated factor with excessive bleeding in HD patients but rather smoking, cardiovascular disease and age [24,33]. In our patients, age was not associated with bleeding.

### Limitations

There are some limitations to our study. First, it is a before-and-after observational study and not a randomized clinical trial thus our conclusions cannot be considered as strong evidence and further larger studies are needed to confirm our findings. Second, it comes from a single center and this might carry a selection bias. Third data on international normalized ratio (INR) measurements were insufficient; INR may have not been always in the therapeutic range and this may explain the absence of hemorrhagic stroke in patients with uncontrolled hypertension on acenocoumadin and the variability in bleeding events in that subgroup. In addition, medications in our study were captured based on prescription, and adherence could not be verified.

Despite those few limitations, our study has the longest follow-up among all cohorts that studied bleeding and mortality in hemodialysis patients simultaneously. It is also the only one that assessed the degree of hypertension severity in HD patients who developed cerebral hemorrhage while on aspirin. Besides, all consecutive patients were recruited into the study and there was no other policy change that could have interfered with the final results.

## Conclusion

This study has gone some way towards enhancing our understanding of bleeding risks in hemodialysis patients. It has shown that aspirin represents a high risk for intracerebral hemorrhage in hemodialysis patients when combined with uncontrolled hypertension. Additionally, we were not able to confirm any beneficial effects of aspirin on the cardiovascular mortality in patients on hemodialysis. Our work has several implications for the daily practice for cardiologists and nephrologists in hemodialysis units. Our results suggest that HD patients who receive aspirin should be thoroughly selected based on hypertension severity. This needs to be confirmed in further larger trials.

## Supporting information

S1 Table Distribution of different bleeding events among different antithrombotic agents’ categories. (DOCX)

S2 Table Yearly inclusion and death of patients between 2005 and 2015. (DOCX)
