# Supplementary material for: Reduction of intracerebral hemorrhage in hemodialysis patients after reducing aspirin use: A quality-assurance observational study
Source: PLoS One. 2017 Oct 2;12(10):e0185847. doi: 10.1371/journal.pone.0185847 (PMC5624631; doi:10.1371/journal.pone.0185847)
Supplement: S1 Table — (DOCX) [file pone.0185847.s001.docx]

| **Phase one** | **Aspirin alone (n=26)** | **Clopidogrel alone (n=15)** | **Dual Antiplatelet**  **(n=4)** | **Acenocoumadin**  **(n=19)** | **None**  **(n=61)** | **Total**  **(n=125)** |
| --- | --- | --- | --- | --- | --- | --- |
| Total Bleeding | 7 | 1 | 1 | 6 | 1 | 16 |
| ICH | 6 | 1 | 0 | 0 | 0 | 7 |
| GIB | 1 | 0 | 1 | 5 | 1 | 8 |
| Other Bleeding Sites | 0 | 0 | 0 | 1 | 0 | 1 |
| **Phase two** | **Aspirin alone (n=11)** | **Clopidogrel alone (n=21)** | **Dual Antiplatelet (n=10)** | **Acenocoumadin (n=12)** | **None (n=56)** | **Total (n=110)** |
| Total Bleeding | 2 | 2 | 2 | 3 | 6 | 15 |
| ICH | 0 | 0 | 0 | 0 | 0 | 0 |
| GIB | 1 | 2 | 1 | 3 | 6 | 13 |
| Other Bleeding sites | 1 | 0 | 1 | 0 | 0 | 2 |
