# Supplementary material for: Reduction of intracerebral hemorrhage in hemodialysis patients after reducing aspirin use: A quality-assurance observational study
Source: PLoS One. 2017 Oct 2;12(10):e0185847. doi: 10.1371/journal.pone.0185847 (PMC5624631; doi:10.1371/journal.pone.0185847)
Supplement: S2 Table — (DOCX) [file pone.0185847.s002.docx]

|  | **Old patients** | **New patients** | **Died** | **Transplanted, transferred or shifted to PD** | **Total followed yearly** | **Yearly mortality** |
| --- | --- | --- | --- | --- | --- | --- |
| **2005** | 30 | 19 | 11 | 4 | 49 | 22.4% |
| **2006** | 34 | 15 | 8 | 2 | 49 | 16.3% |
| **2007** | 39 | 20 | 12 | 0 | 59 | 20.3% |
| **2008** | 47 | 8 | 5 | 1 | 55 | 9% |
| **2009** | 49 | 13 | 5 | 1 | 62 | 8% |
| **2010** | 56 | 10 | 7 | 1 | 66 | 10.6% |
| **2011** | 58 | 10 | 12 | 3 | 68 | 17.6% |
| **2012** | 53 | 13 | 7 | 0 | 66 | 10.6% |
| **2013** | 59 | 18 | 10 | 0 | 77 | 13% |
| **2014** | 67 | 14 | 13 | 2 | 71 | 18.3% |
| **2015** | 56 | 12 | 11 | 1 | 68 | 16.1% |
